# Perioperative echocardiography-guided hemodynamic therapy in high-risk patients: a practical expert approach of hemodynamically focused echocardiography

**DOI:** 10.1007/s10877-020-00534-7

**Published:** 2020-05-26

**Authors:** R. F. Trauzeddel, M. Ertmer, M. Nordine, H. V. Groesdonk, G. Michels, R. Pfister, D. Reuter, T. W. L. Scheeren, C. Berger, S. Treskatsch

**Affiliations:** 1 Department of Anesthesiology and Intensive Care Medicine, Campus Benjamin Franklin, Charité - Universitätsmedizin Berlin, Corporate Member of Freie Universität Berlin, Humboldt-Universität zu Berlin, and Berlin Institute of Health, Berlin, Germany; 2 Department of Anesthesiology, Unfallkrankenhaus Berlin, Berlin, Germany; 3 Department of Interdisciplinary Intensive Care Medicine and Intermediate Care, Helios Hospital Erfurt, Erfurt, Germany; 4 Department of Internal Medicine III, Heart Center, Faculty of Medicine and University Hospital Cologne, University of Cologne, Cologne, Germany; 5 Department of Anesthesiology and Intensive Care Medicine, University of Rostock, Rostock, Germany; 6 Department of Anesthesiology, University Medical Center Groningen, University of Groningen, Groningen, Netherlands

**Keywords:** Perioperative, Echocardiography, Hemodynamic optimization, Monitoring

## Abstract

The number of high-risk patients undergoing surgery is growing. To maintain adequate hemodynamic functioning as well as oxygen delivery to the vital organs (DO_2_) amongst this patient population, a rapid assessment of cardiac functioning is essential for the anesthesiologist. Pinpointing any underlying cardiovascular pathophysiology can be decisive to guide interventions in the intraoperative setting. Various techniques are available to monitor the hemodynamic status of the patient, however due to intrinsic limitations, many of these methods may not be able to directly identify the underlying cause of cardiovascular impairment. Hemodynamic focused echocardiography, as a rapid diagnostic method, offers an excellent opportunity to examine signs of filling impairment, cardiac preload, myocardial contractility and the function of the heart valves. We thus propose a 6-step-echocardiographic approach to assess high-risk patients in order to improve and maintain perioperative DO_2_. The summary of all echocardiographic based findings allows a differentiated assessment of the patient's cardiovascular function and can thus help guide a (patho)physiological-orientated and individualized hemodynamic therapy.

## Background

Adequate oxygen delivery (DO_2_) is of utmost importance for the maintenance of homeostatic organ function and is significantly dependent upon cardiac stroke volume (SV). Determinants of SV are pre- and afterload, intrinsic contractility, heart rate/rhythm as well as cardiac valve function. It has long been known that a critically reduced DO_2_ can worsen the perioperative outcome by promoting a systemic inflammatory response (SIRS) and organ dysfunction through hypoperfusion [1, 2]. High-risk patients, with or without pre-existing cardiac disease, may have an increased risk for a compromised SV during the perioperative period and demand a specific level of monitoring [3].

Extensive research has shown that perioperative hemodynamic optimization amongst high-risk patients can reduce post-operative complications [4–10]. Various advanced—and mostly invasive—hemodynamic monitoring techniques are available in daily clinical practice [11], however, particular clinical circumstances (e.g. arrhythmia, right ventricular dysfunction, lung-protective or one-sided ventilation) limit the reliability of some of these techniques, e.g. SV measurement, and pulse pressure variation (PPV). The main advantage of these hemodynamic monitoring techniques is the ability to measure important surrogate variables for cardiovascular function over time. This allows for a continuous evaluation of the effect of therapeutic interventions such as fluid substitution or vasoactive medication administration. The main disadvantages of these monitoring techniques, however, is the inability to directly assess overall intravascular fluid status and the cardiovascular cause of a reduced DO_2_ [12]. For example, a reduced SV can be caused by hypovolemia, reduced LV systolic function or pericardial tamponade, all of which require differing intervention strategies in order to maintain hemodynamic stability. Furthermore it has been specifically shown that arterial blood pressure and SV do not have a linear relationship with one another [13], thereby negating an exclusive reliance upon arterial blood pressure as an indicator of DO_2_.

In this context, transthoracic (TTE) and transesophageal (TEE) echocardiography are becoming increasingly essential for the anesthesiologist [14–16]. Echocardiography-guided hemodynamic examination provides a real-time pathophysiological-oriented approach, which allows for the evaluation of both left and right cardiac function and the relative circulatory state [17]. It has been shown that use of an echocardiography-based hemodynamic optimization protocol improved outcomes amongst septic patients in an ICU (Intensive Care Unit) setting [18–20]. In hemodynamically unstable patients unresponsive to initial treatment, there is a class I indication for performing a timely echocardiographic examination in order to accurately assess and implement interventions aimed at maintaining hemodynamic stability [21–25]. Interestingly, it has been shown that a hemodynamically focused echocardiography seems to be sufficient in guiding cardiovascular therapy [25–29]. Nevertheless, experience is essential in order to adequately interpret and evaluate TTE/TEE findings. Therefore, a standardized curricular training based on pathophysiological hemodynamic issues should be implemented in order to uphold quality practice standards [30], as well as available standard algorithms for performing TTE/TEE [31]. There is evidence to suggest, however, that after an initial 2-h TTE training course, anesthesiologist without prior experience in echocardiography could obtain adequate image via TTE compared with cardiothoracic anesthesiologist fellows [32], yet interpretation of the clinical scenario and the necessary interventions needed require a certain level of expertise. “Focused examiners” have the responsibility to *seek expert help* whenever needed. In this context, continuously available supervision by physicians with curricular training and certification in hemodynamics and echocardiography in the field of anesthesiology/intensive care/cardiology has to be ensured.

In this article, a practical step-by-step approach towards a perioperative echocardiographic-based hemodynamic optimization for high-risk surgical patients is presented. It should be noted that the proposed algorithm may be used as a diagnostic tool “as needed”, e.g. patients presenting with hemodynamic instability or signs of hypoperfusion, or “as a predefined monitoring tool” within a goal-directed treatment strategy, e.g. major abdominal/vascular surgery. In the latter case physicians have to set monitoring intervals which they think may appropriately address the patient´s hemodynamic risk as echocardiography is a discontinuous method. Here, frequency of echocardiographic evaluations determines the ability to optimize hemodynamics. In addition, using the proposed algorithm as a predefined monitoring tool, it may be beneficial being able to compare recent echocardiographic findings intra-/postoperatively with a preoperative baseline exam. It should be noted that possible hemodynamic relevant echocardiographic findings must always be interpreted while integrating the clinical situation along with the patients’ medical background.

## Main text

In order to properly perform a focused echocardiography, the following views should be used: (1) *TTE*: parasternal long (PLAX) and short axis (PSAX), apical (4C) or subcostal 4-chamber view (S4C) with subcostal view of the inferior vena cava (SIVC); (2) *TEE*: midesophageal 4-chamber view (ME4C), midesophageal view of the superior vena cava (SVC), transgastric short axis view (TGSAX) [33]. In addition to the two-dimensional echocardiographic evaluation, the use of (color) doppler modalities may allow for limited qualitative evaluation of the heart valves [34]. Both TTE and TEE analysis are applicable, however, TEE may offer overall better image quality, particularly if lungs are mechanically ventilated or a transthoracic/subcostal approach is not feasible, e.g. lung surgery. It may also be preferable in patients who presenting with obesity. The major drawback of the TEE approach is a higher invasiveness, along with a longer “set-up” time. Non-invasive TTE may be more practical, especially in non-cardiac surgery cases and in ICU, where a rapid diagnostic is needed in the event of hemodynamic instability or as a (preoperative) screening tool (“baseline exam”) [35]. To our knowledge, no study has directly compared the efficiency of TTE with TEE with regards to their respective effectiveness in determining intra-operative cardiac function amongst high-risk patients. Therefore no data exist on the preference of one technique over the other, and we leave that choice up to the clinician involved in the case. Nevertheless, image acquisition will be impossible in some patients at all as well as in most patients in prone position.

### Step 1: Evaluation of "Cardiac filling impairment"

**Fig. 1:**
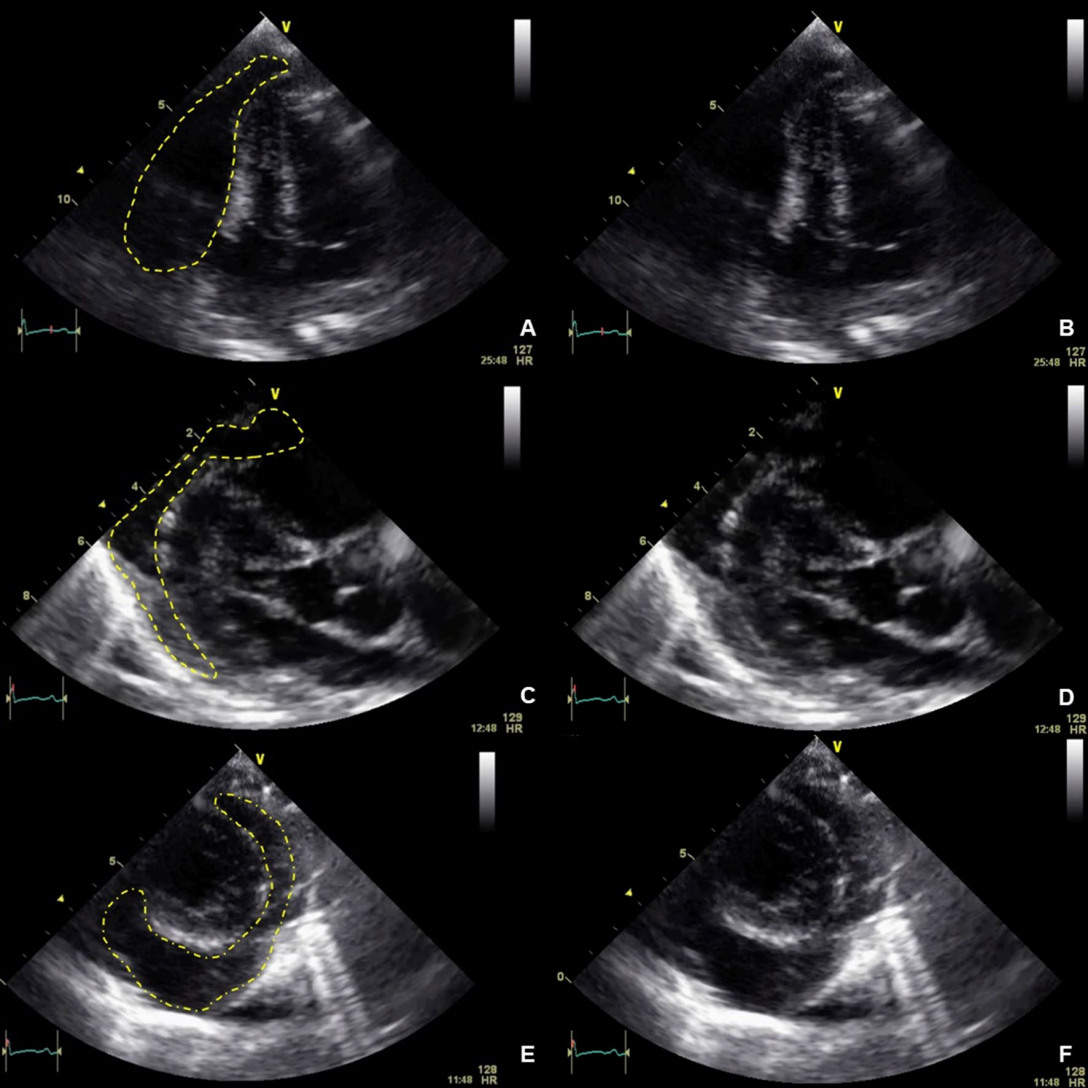
Pericardial tamponade. **a** Highlighted in yellow, via 4C view. **b** Without highlights, via 4C view. **c** Highlighted in yellow, via PLAX view. **d** Without highlights, via PLAX view. **e** Highlighted in yellow, via PSAX view. **f** Without highlights, via PSAX view

If cardiac filling is impaired by pericardial effusion/tamponade as shown in Fig. 1 ("obstructive shock"), evacuation (interventional or surgical) has the highest priority. Not only in the cardiac surgery setting, but also due to trauma or due to chronic disease, a relevant accumulation of fluid in the pericardium can occur. Within the "Focused Assessment with Sonography in Trauma" algorithm (FAST) the orienting visualization of all four heart chambers with the possibility of visualizing pericardial effusion is therefore an integral part of initial trauma assessment [36–39]. Echocardiographic signs of hemodynamically relevant pericardial effusions with a given clinical history and/or symptomatology may include: identification of pericardial effusion with consecutive hypovolemia of all heart chambers, collapse of the right cardiac chambers and/or dilatation of the inferior vena cava (SIVC). When using a TTE, the S4C view should be used, while for TEE, the ME4C should be used initially.

### Step 2: Evaluation of "Volume status/responsiveness”

Once any immediate impairment of cardiac filling has been ruled out, the second step is to estimate the volume status/responsiveness of the patient, as both hypo- and hypervolemia can reduce SV and thus DO_2_. To assess the volume status, the 4-chamber views (4C) as well as the short axis views (SAX) at the level of the papillary muscles are suitable for obtaining a quick overview.

**Fig. 2:**
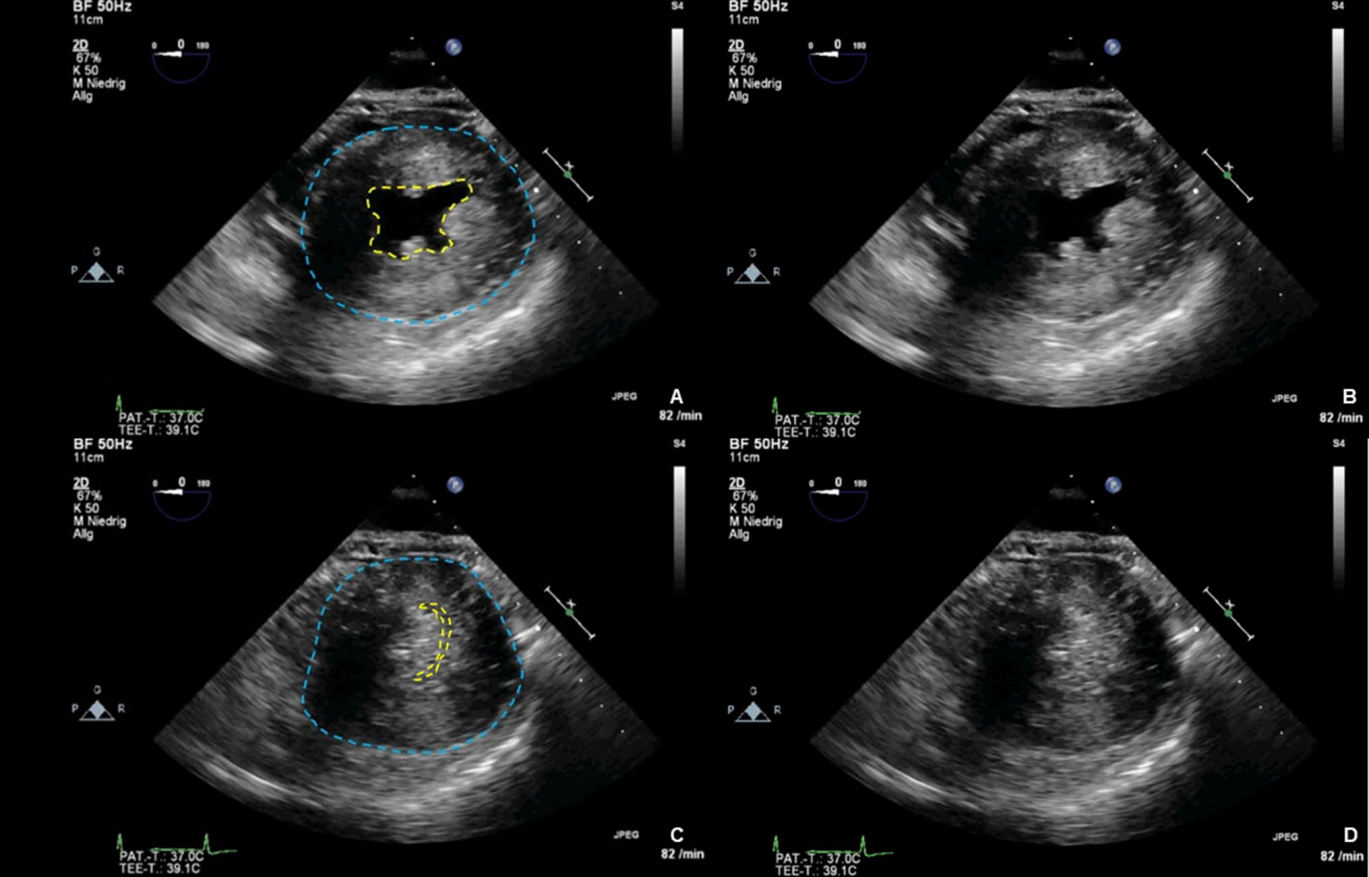
Concentric hypertrophy. **a** End diastolic, with endocardium highlighted in yellow and epicardium highlighted in blue, via PSAX view. **b** End diastolic, without highlights, via PSAX view. **c** End systolic, with epicardium highlighted in blue, via PSAX view. **d** End systolic, without highlights, via PSAX view

Although resting diameters for cardiac chambers are gender and body surface area specific [40], the size of the left ventricle (LV) and the right ventricle (RV) should be measured with regards to overall volume status. An end-diastolic left ventricular diameter (LVEDD) of 35–55 mm may reflect normal LV and a basal RV diameter ≤ 41 mm may reflect normal RV size. Qualitatively, substantial hypovolemia may be identified by the “kissing papillary muscle” sign of the corresponding ventricle. This sign is best witnessed during the systolic period, whereby the opposite myocardial walls of the associated ventricle come in contact with one another. Occasionally, hypovolemia will aggravate a dynamic flow obstruction in the left ventricular outflow tract (LVOT) in case of LV hypertrophy. It should be noted, that a pronounced concentric hypertrophy as evidenced by a myocardial wall thickness of > 14 mm (i.e., due to severe aortic stenosis or as primary disease as displayed in Fig. 2) must be excluded prior to the diagnose of hypovolemia [41]. In addition, a preoperative dilated LV (e.g. LVEDD 65 mm) with a reduced global systolic function may be interpreted as “hypovolemic” if the LVEDD is within normal range (e.g. LVEDD 50 mm) and DO_2_ is reduced.

**Fig. 3:**
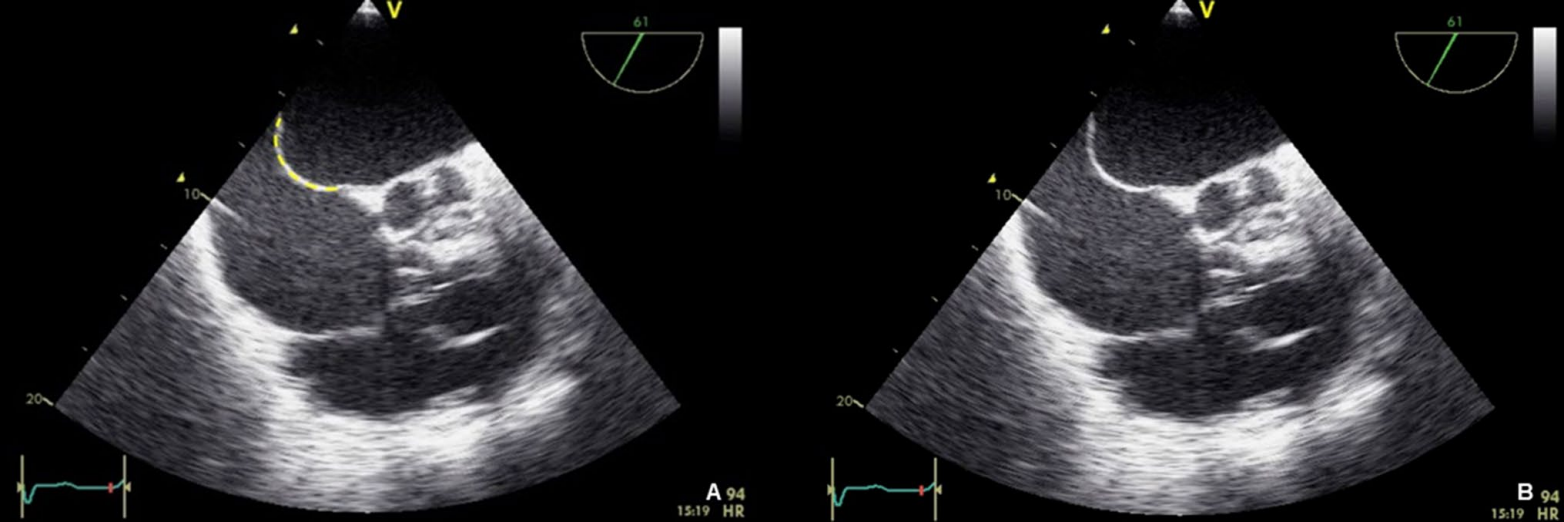
Right convex interatrial septum. **a** Septum highlighted in yellow, via 4C view. **b** without highlights, via 4C view

With regards to atrial volume status, a visual assessment of the interatrial septum (IAS) in the 4-chamber views (4C, ME4C) can be used for qualitative estimation of atrial filling pressures. During states of low bi-atrial filling such as during global hypovolemia, a hypermobile IAS is commonly observed. With increasing left atrial filling pressure, the IAS appears permanently convex to the right (as displayed in Fig. 3), whereas with increased right atrial filling pressure, the IAS appears permanently convex to the left atrium in combination with left cardiac hypovolemia [42]. In the context of global hypervolemia, all heart chambers appear "overfilled” or “stretched" and the IAS is usually fixated in the middle [43, 44].

**Fig. 4:**
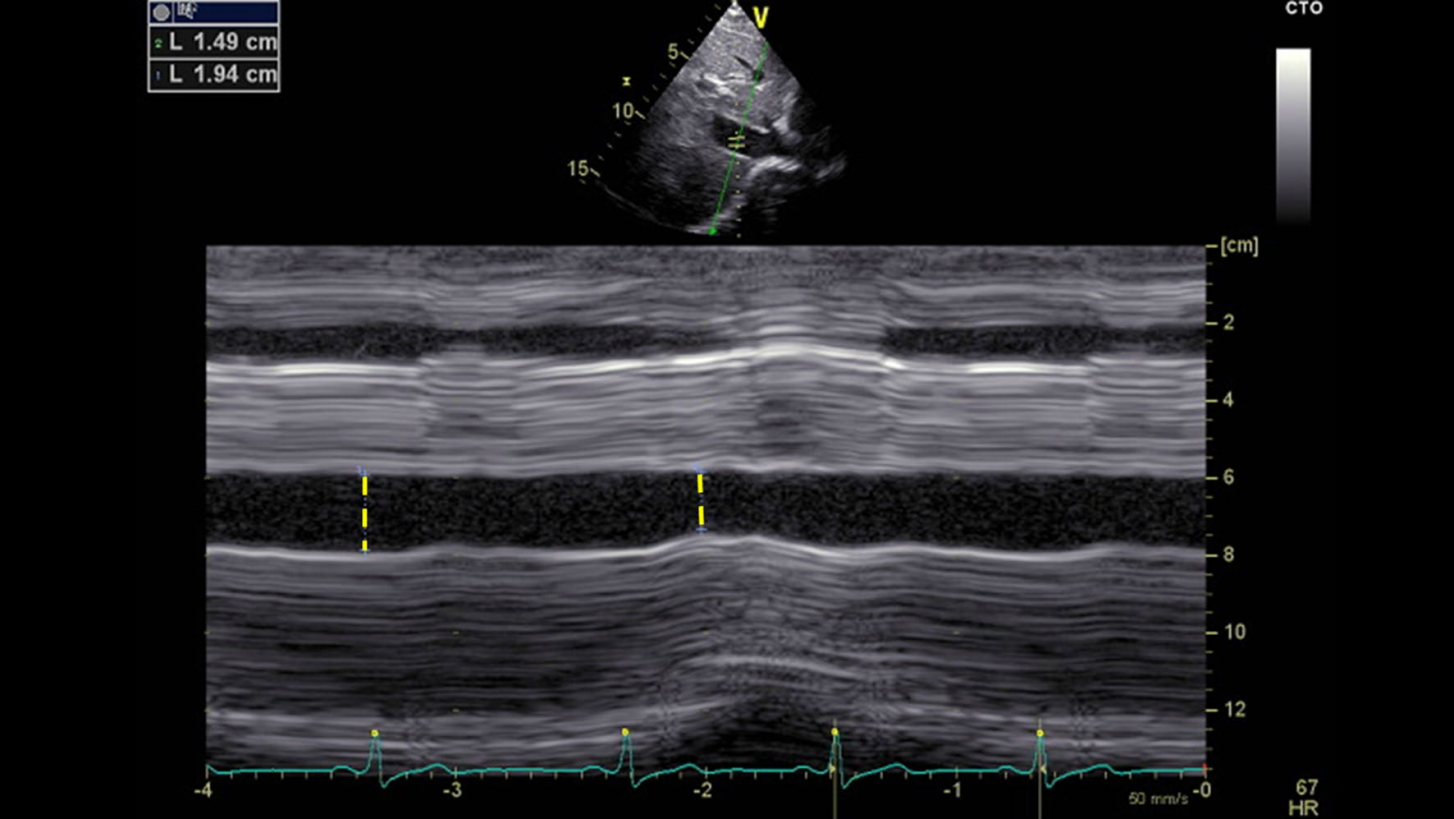
Inferior vena cava via TTE. Marked in yellow is the diameter with measurements given

Volume status/responsiveness can be estimated by measuring the superior vena cava (SVC) via TEE or the inferior vena cava (IVC) via TTE as shown in Fig. 4. The SIVC diameter and its respiratory variation may be used to estimate right atrial filling pressure [45]. The normal diameter for the SIVC is < 21 mm in awake and spontaneously breathing patients [46]. Due to the increased intrathoracic pressure exerted during mechanical inspiration, venous return is reduced and the IVC distends ("SIVC-Distensibility index, DI") [47]. The more pronounced the intravascular hypovolemia, the greater the volume responsiveness, thus the greater the IVC distensibility [48]. An SIVC-DI of > 18% in controlled ventilated septic patients indicated a positive volume response with an increase in cardiac output (CO) after fluid resuscitation [49–53]. In patients with preserved spontaneous respiration, sufficient sensitivity and specificity of the SIVC-DI can also be achieved [54]: the patient is asked to inhale deeply once and exhale passively afterward, while an ultrasound measurement is continuously recorded. An SIVC diameter variability of ≥ 48% represents a positive volume responsiveness. The same is also possible with TEE using the SVC collapse index (SVC-CI) [55]. Due to the intrathoracic position, the SVC will be compressed during mechanical inspiration. Here, a SVC-CI > 36% indicates a positive volume responsiveness. However, like many other methods, these easy-to-determine quantitative variables are subject to individual cut-off variations (e.g. SIVC-DI "grey zone" 8—30%) [53, 56–59]. Therefore, in addition to the quantitative determination of these two indices, the approach shown in Table 1 may be helpful in deciphering the measurements taken from the SIVC/SVC [60–62]. Again, physicians have to interpret echocardiographic findings in the clinical context: fluid substituon will be indicated in a trauma patient with low blood pressure, overall small heart chambers and a small vena cava inferior.

**Table 1:** Qualitative echocardiographic evaluation of volume status / fluid responsiveness

Status	Respiratory Modulation	Interpretation	Fluid responsiveness
SIVC/SVC dilated *(i.e. round in shape, stretched, visual aspect of overfilling)*	No variation	Filling pressure ⇧	Negative *(“Stop signal” for further fluid administration)*^a^
SIVC/SVC small/collapsed	Pronounced variation	Filling pressure ⇩	Positive
SIVC/SVC intermediate	Passive Leg Raising (PLR) and/or Fluid challenge (FC) If stroke volume increases with unchanged systemic resistance, fluid substitution is clinically indicated		

^a^In the context of chronic cardiovascular disease, a positive volume responsiveness may occasionally be given despite a dilated SIVC without respiratory oscillation. Further evaluation may be done by means of PLR/FC

Taken together from a clinical point of view, one has to differentiate between (a) “global” hypovolemia (i.e. all heart chambers are reduced in size due to a significant reduction in total circulating blood volume—additional fluid substitution will lead to an increase in SV), (b) “relative” hypovolemia (i.e. all heart chambers appeared to be “normally” filled, however, additional fluid substitution may cause an increase in SV—“volume responsiveness”) or (c) “partial” hypovolemia (i.e. LV hypovolemia in case of RV failure). In the latter, fluid substitution will mostly not be effective in increasing left ventricular SV because of the incapability of the RV to transport the blood forward into the pulmonary circulation and left heart, thus worsening RV cogestion. The determination of the exact hypovolemic cause will be detrimental in defining the amount and type of fluid resuscitation necessary.

### Step 3: RV evaluation

A restricted RV function is associated with increased perioperative mortality [63–65]. In addition, as already mentioned, a sufficient LV function depends on a sufficient preload provided by the RV [66]. Therefore, the morphology and function of the RV should be assessed prior to LV assessment [67].

**Fig. 5:**
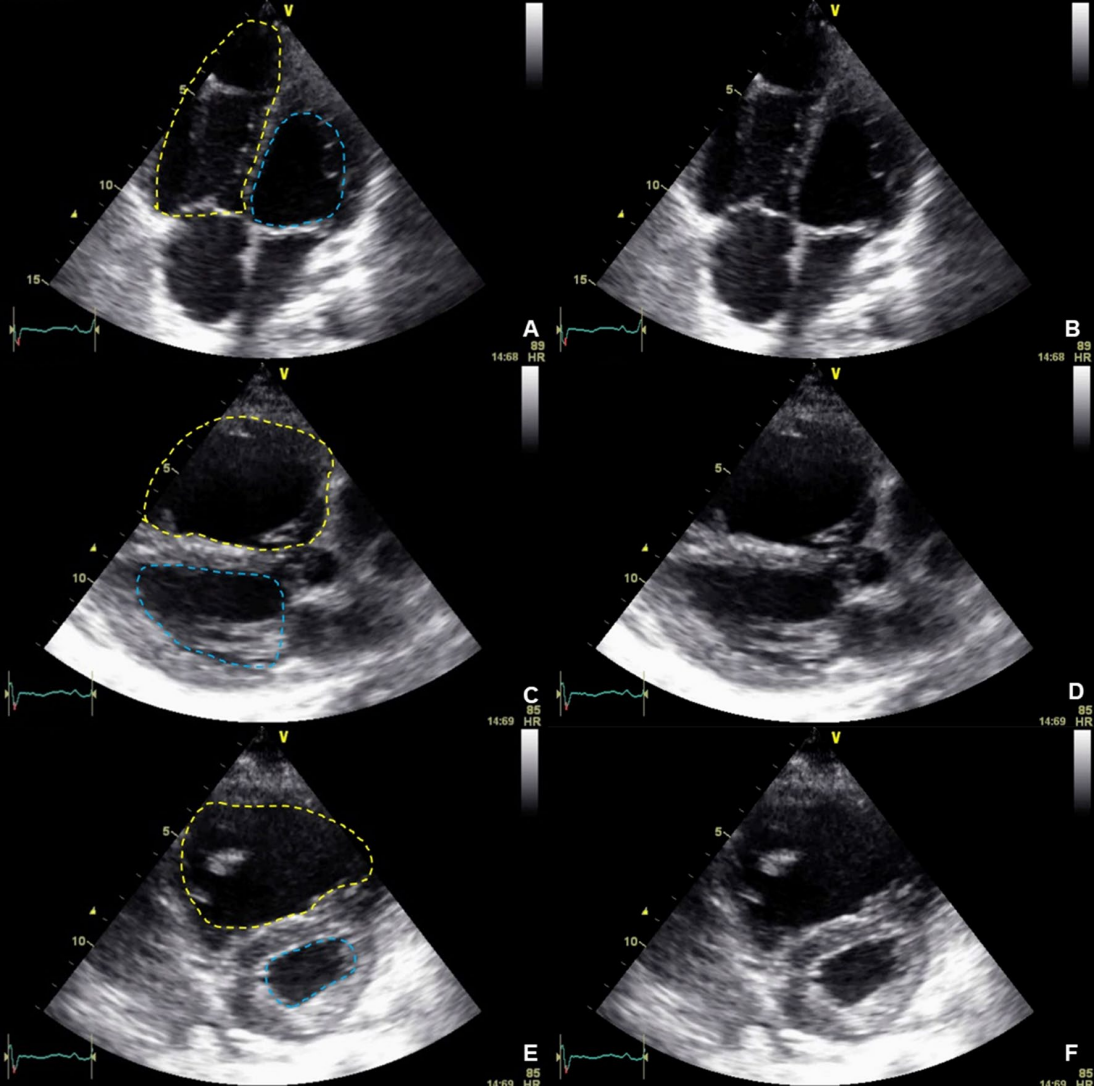
Right heart dilation. **a** with right ventricle highlighted in yellow and left ventricle highlighted in blue, via 4C view. **b** without highlights, via 4C view. **c** with right ventricle highlighted in yellow and left ventricle highlighted in blue, via PLAX view. **d** without highlights, via PLAX view. **e** with right ventricle highlighted in yellow and left ventricle highlighted in blue, via PSAX view. **f** without highlights, via PSAX view

In addition to the points mentioned in step 2, this is achieved visually with the help of the volume/diameter relation between the right and left ventricle, the "RV/LV-Index". A normal ratio is ~ 0.6, an RV/LV index ≥ 1.0 indicates a severe RV dilatation as shown in Fig. 5 [68].

In case of RV dysfunction, hypertrophy of the free right ventricular wall (> 5 mm) may indicate a chronic disease process [69]. The thickness of the right ventricular wall is best measured from subcostal at the level of the anterior tricuspid valve tip under recess of trabeculae and papillary muscles. Alternatively, measurement of the thickness of the right ventricle may be performed in the PLAX [45].

**Fig. 6:**
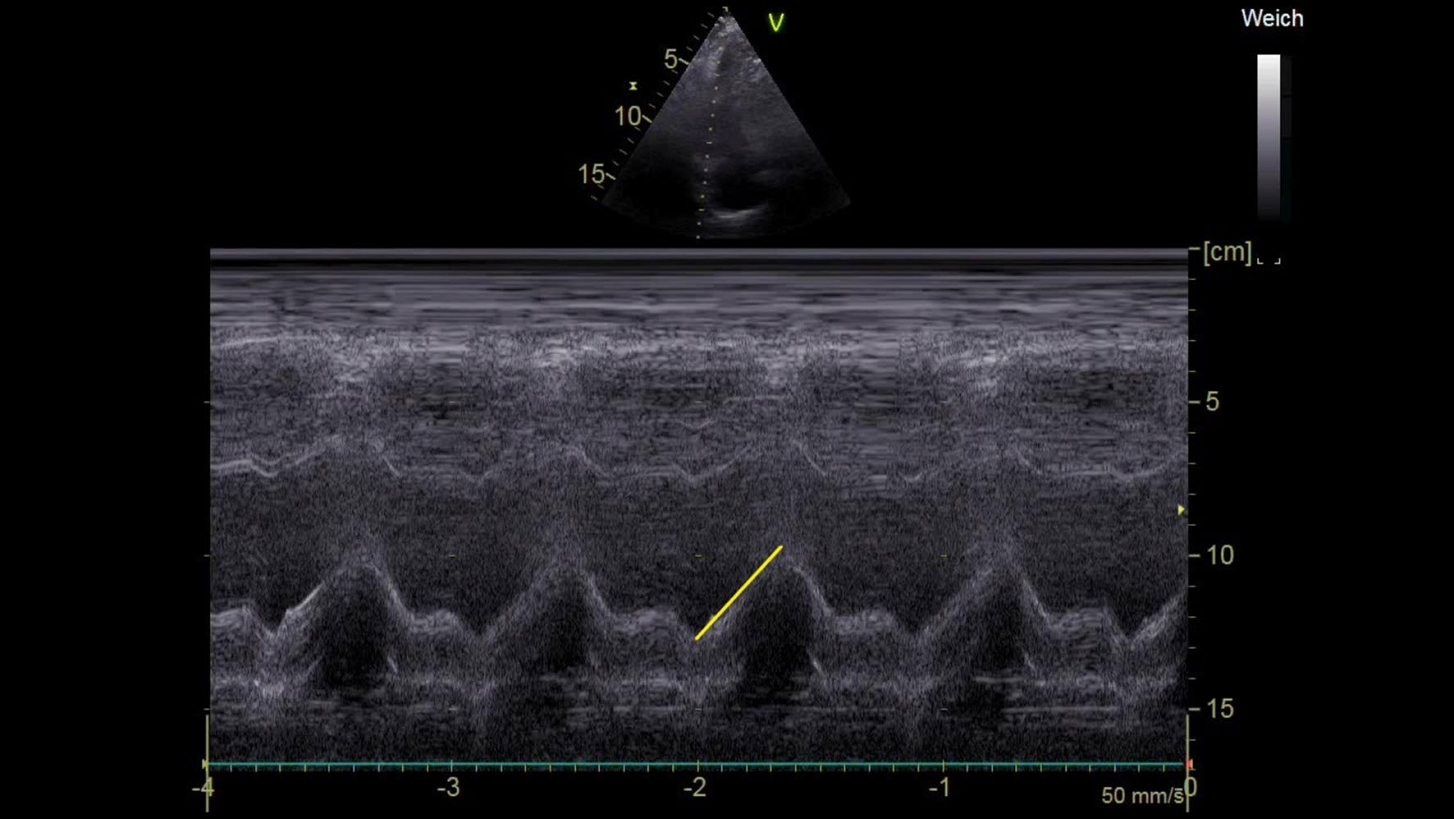
TAPSE, with tricuspid annulus excursion marked in Motion-Mode

Contractility of the RV is visually assessed in the 4-chamber views. With a normal RV function, the free RV wall should move inwards [45]. For simple quantitative evaluation of the RV function, the amount of systolic movement of the lateral tricuspid valve annulus towards the apex (Tricuspid Annular Plane Systolic Excursion, TAPSE) can be used (Fig. 6). A TAPSE of ≥ 17 mm indicates normal systolic RV function [45]. If RV dilatation and systolic impairment are observed, this mostly reflects severe, and hemodynamic relevant RV dysfunction.

In addition to advanced diagnostics and consecutive therapy of the primary cause of RV dysfunction (e.g. lysis in pulmonary embolism or revascularization in RV infarction), hemodynamic optimization should aim at optimizing RV preload, ensuring coronary perfusion pressure, as well as inotropic support and pulmonary afterload reduction if indicated [67, 70–73]. Importantly, optimizing RV preload must be performed with great caution to avoid volume overload. RV volume overload is detrimental not only for the contractile function of the RV, but also for coronary perfusion, venous, and intramural perfusion pressure of other organs such as the kidney. Furthermore, LV output is dependent upon the physiological geometry of the RV and the septum. Hence, RV overloading can displace the interventricular septum towards the LV (“paradoxical septum shift”), thereby restricting LV contractility. If the required therapeutic interventions are not successful, extracorporeal support—if available—may be considered [72, 74]. If the RV is assessed as "non-dilated, normal systolic function", hemodynamically relevant RV dysfunction is excluded and one can proceed to step 4.

### Step 4: LV evaluation

**Fig. 7:**
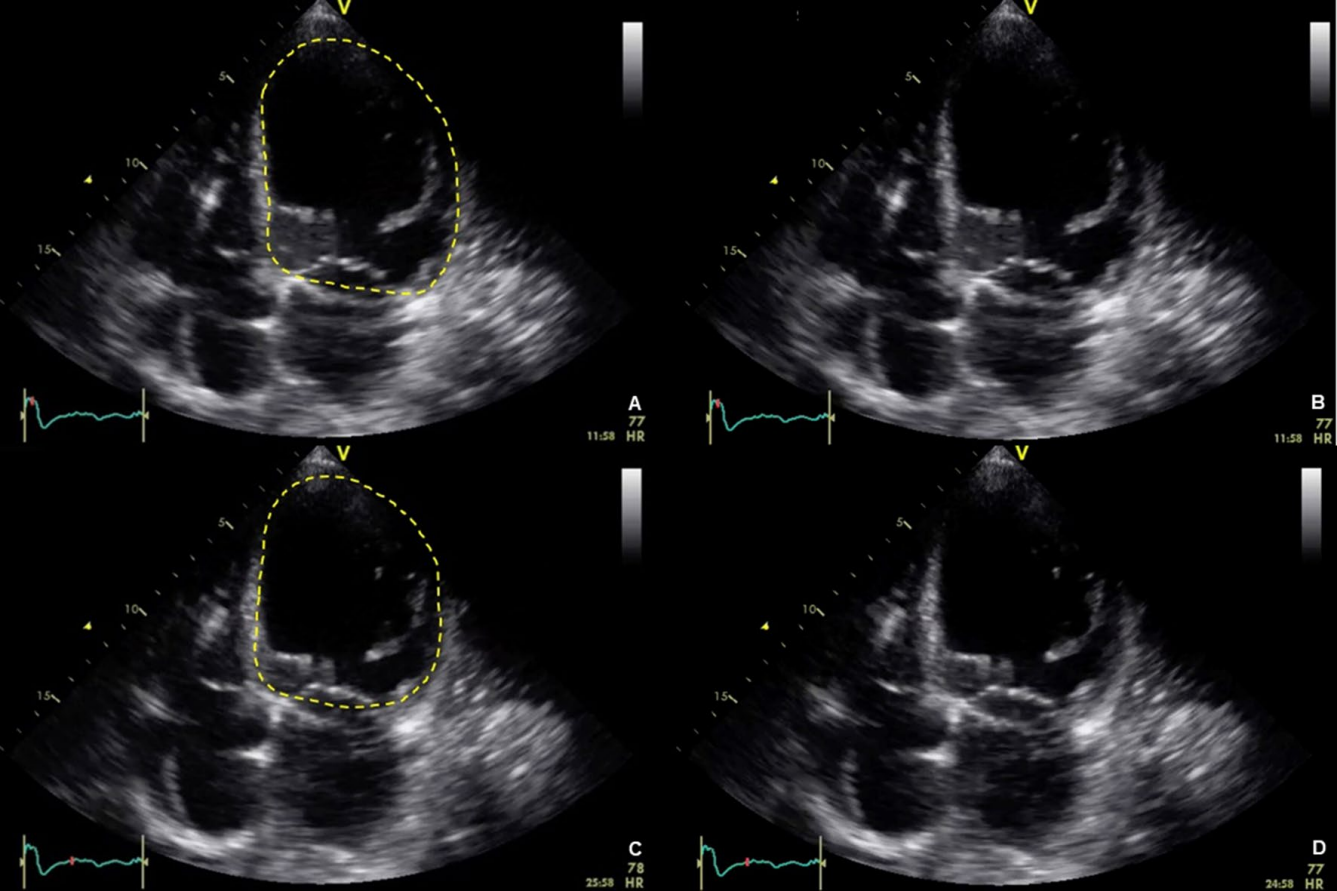
Left ventricular dysfunction. **a** End diastolic phase, left ventricle highlighted in yellow, via 4C view. **b** End diastolic phase, without highlights, via 4C view. **c** Dilation in end systolic phase, left ventricle highlighted in yellow, via 4C view. **d** Dilation in end systolic phase, without highlights, via 4C view

In the fourth step, the LV should now be assessed in an analogous manner to the RV with regard to size and global systolic function (see also steps 2 and 3). The left ventricular ejection fraction (LVEF) is determined to quantify global systolic function. For normal clinical concerns, however, a qualitative assessment of the LVEF ("eye balling") may be equivalent to a quantitative [75]. The transthoracic parasternal short axis view (PSAX) or the transgastric central papillary short axis view (TGSAX) as well as 4-chamber views (4C or ME4C) allow for a quick orientation (Fig. 7) [76].

If the LV appears non-dilated with normal systolic function (LVEF > 50%), relevant systolic LV dysfunction is excluded. However, isolated diastolic LV dysfunction (Heart failure with preserved ejection fraction, HFpEF) may be present, thereby affecting overall hemodynamic functioning [77]. Evaluation of diastolic function is outside of the scope of a hemodynamic focused echocardiography. If diastolic dysfunction is suspected, an expert consultation should be made in order to guide further diagnostics and therapy [78]. Qualitatively, a pronounced dilation of the left atrium (LA) in conjunction with a “stiff” and/or hypertrophied LV with normal systolic function in a breathless patient may be related to HFpEF [79]. In symptomatic patients LV afterload should be reduced and fluid substitution should be restricted [24, 80].

In the case of a non-dilated LV with slightly to moderately reduced global systolic function (heart failure with mid-range ejection fraction (HFmrEF), LVEF 40–49%), cardiac preload should be optimized and inotropic support may be administered to improve DO_2_ [74, 81]. In a dilated LV with severely reduced global systolic function (heart failure with reduced ejection fraction (HFrEF) with LVEF < 40%) an intensified inotropic therapy in conjunction with preload optimization is indicated in situations of hemodynamic instability. A vasopressor may be considered in case of cardiogenic shock with persistent hypoperfusion, despite treatment with an inotropic agent, to increase blood pressure and vital organ perfusion pressure [24]. If conservative therapy does not improve DO_2_, mechanical support and/or implantation of a left ventricular microaxial pump may be discussed. Lastly, if (new) regional wall motion abnormalities are detected, specifically LV wall hypokinesia, akinesia or dyskinesia [82], this may hint at specific cause such as myocardial infarction or Takotsubo syndrome, which require specific diagnostic testing (e.g. electrocardiogram, cardiac enzymes, coronary angiography) and treatment.

### Step 5: Evaluation of „Valve morphology and function“

**Fig. 8:**
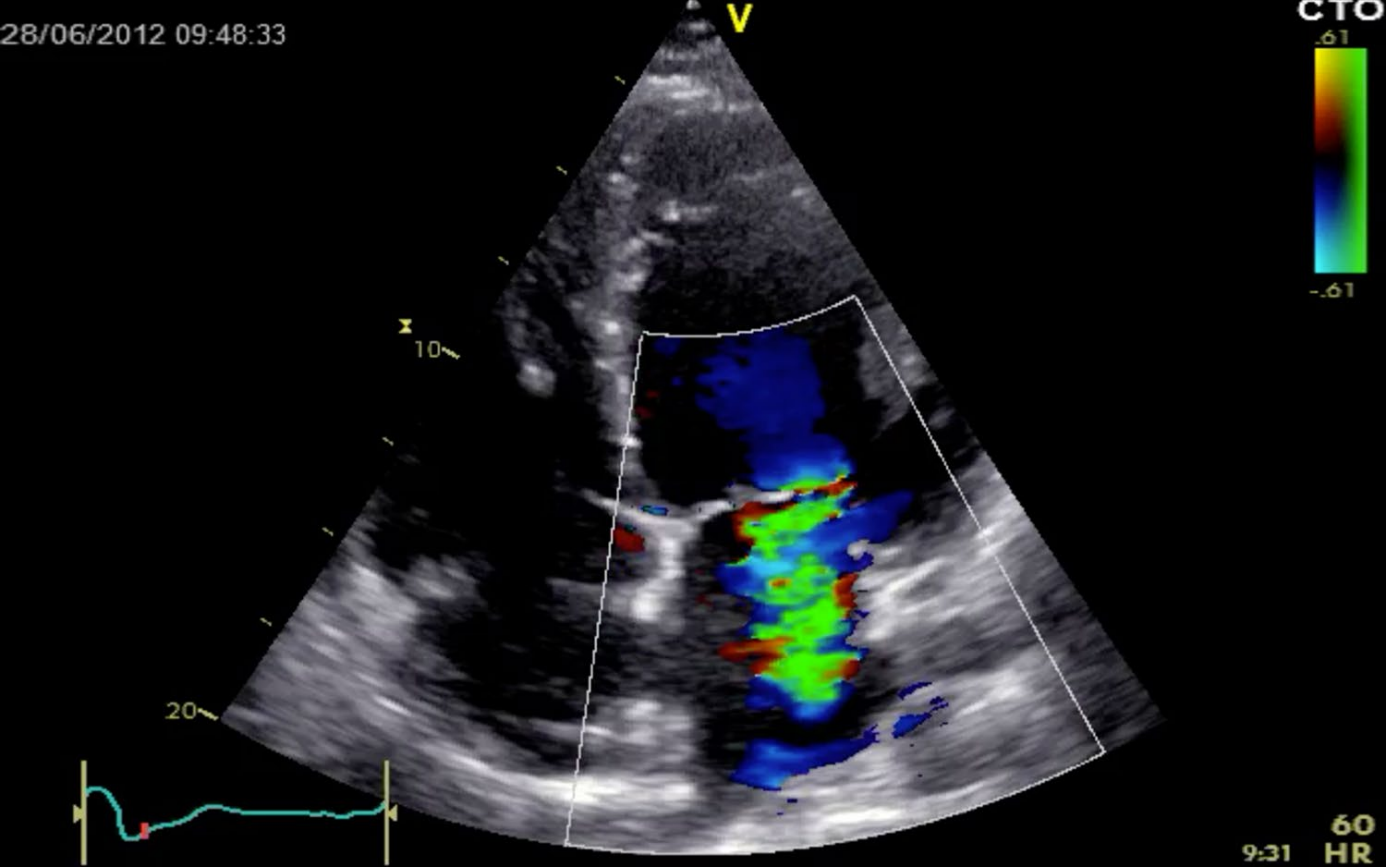
Mitral valve regurgitation, with doppler, via 4C view

Echocardiography allows for a comprehensive morphological and functional assessment of the heart valves. The visual and thus qualitative evaluation of valves in the hemodynamic focused examination is used to assess valve opening and closure as well as to recognize morphological abnormalities. Hemodynamic relevant valve dysfunction may be excluded if thin leaflets with a normal opening/closing and without turbulent flow in color Doppler have been determined in ≥ 2 cross-sectional views. If a thickened or calcified valve with a restricted opening is apparent, hemodynamic relevant stenosis may be suspected, especially in the case of antegrade flow accelerations/turbulences in color Doppler. In addition, hemodynamic relevant regurgitation might be suspected if an exaggerated leaflet motion or visuable coaptation defect during valve closing is observed in conjunction with a wide, turbulent colour jet (“vena contracta”) depicting significant backward flow (Fig. 8) [34]. However, in case of hemodynamically relevant valve abnormalities in the focused examination, a detailed evaluation should be carried out immediately by a certified examiner [83–85].

### Step 6: Rating cardiac output

**Fig. 9:**
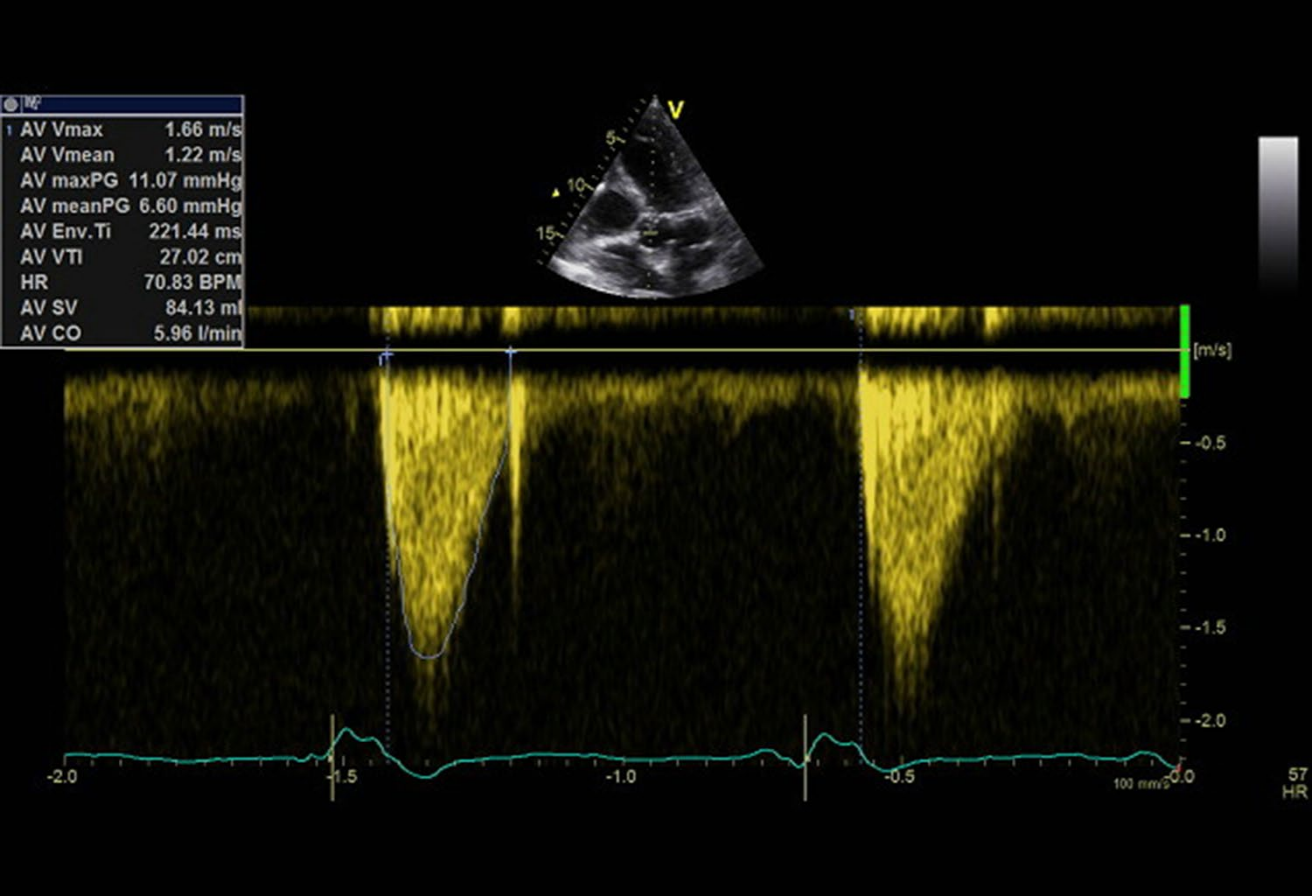
Continouous wave Doppler across the aortic valve to measure the velocity time integral

Transthoracic and transesophageal echocardiography are capable of rating cardiac output, although discontinuously, using continuous-wave (cw) Doppler across the left ventricular outflow tract (LVOT) / aortic valve (AV) measuring the velocity time integral (VTI) (Fig. 9) [86]. Prior to this, aortic stenosis must be excluded (see Step 5). A VTI of 18–22 cm indicates normal stroke volume, whereas a VTI < 18 cm is suspective of decreased stroke volume and > 22 cm of an increased one [86]. In a prospective observational study Mercado et al. found out that in critically ill mechanically ventilated patients the transthoracic echocardiography was an accurate and precise method for estimating cardiac output [87]. In contrast, amongst patients undergoing cardiac surgery, echocardiography is not interchangeable with cardiac output monitoring by pulmonary catheter thermodilution [88]. Thus, after and/or simultaneously to initial echocardiographic evaluation, a continuous hemodynamic monitoring should be implemented in hemodynamic unstable patients to assess therapeutic success. In patients with refractory shock associated with a right ventricular dysfunction, a pulmonary artery catheter in addition to echocardiography is recommended [89]. Most other conditions may be monitored by transpulmonary thermodilution [90].

## Conclusions

Hemodynamic focused echocardiography as a rapid diagnostic method, offers an excellent opportunity to examine signs of filling impairment, cardiac preload, myocardial contractility and the function of the heart valves. We thus suggest a 6-step-echocardiohgraphic approach to assess high-risk cardiac patients with in the perioperative setting to rapidly pinpoint intra-cardiac pathophysiology. In conclusion, the summary of all echocardiographic findings, including clinical symptoms, allows for a differentiated assessment of patient's cardiovascular function and can thus help to guide a (patho)physiological-orientated and individualized hemodynamic therapy in order to optimize/maintain SV.

## Data Availability

Not applicable

## Abbreviations

4C: 4-Chamber view
AS: Aortic stenosis
AV: Aortic valve
CO: Cardiac output
DO2: Oxygen delivery
FAST: Focused Assessment with Sonography in Trauma
HFmrEF: Heart failure with mid-range ejection fraction
HFpEF: Heart failure with preserved ejection fraction
HFreF: Heart failure with reduced ejection fraction
IAS: Interatrial septum
ICU: Intensive Care Unit
IVC: Inferior vena cava
LA: Left atrium
LV: Left ventricle
LVEDD: End-diastolic left ventricular diameter
LVEF: Left ventricular ejection fraction
LVOT: Left ventricular outflow tract
ME: Midesophageal
PLAX: Parasternal long axis
PPV: Pulse pressure variation
PSAX: Parasternal short axis
RV: Right ventricle
S4C: Subcostal 4-chamber view
SAX: Short axis
SIVC: Subcostal view of the inferior vena cava
SIVC-DI: SIVC-Distensibility index
SV: Stroke volume
SVC: Superior vena cava
SVC-CI: SVC collapse index
TAPSE: Tricuspid Annular Plane Systolic Excursion
TEE: Transesophageal echocardiography
TGSAX: Transgastric short axis
TTE: Transthoracic echocardiography
VTI: Velocity time integral
